# Health education and postpartum depression: exploring associations in clinical data

**DOI:** 10.3389/fpsyt.2025.1627853

**Published:** 2025-09-05

**Authors:** Xianfang Song, Zhengli Kang, Manting Sha, Sha Wang

**Affiliations:** ^1^ Department of International, Shijiazhuang Obstetrics and Gynecology Hospital, Shijiazhuang, Hebei, China; ^2^ The Five Obstetric Ward, Shijiazhuang Obstetrics and Gynecology Hospital, Shijiazhuang, Hebei, China; ^3^ The Seventh Obstetric Ward, Shijiazhuang Obstetrics and Gynecology Hospital, Shijiazhuang, Hebei, China; ^4^ Departments of Emergency, Shijiazhuang Obstetrics and Gynecology Hospital, Shijiazhuang, Hebe, China

**Keywords:** birth, health education, Edinburgh post-birth depression scale (EPDS), state-trait anxiety inventory (STAI), mental state

## Abstract

**Background:**

Postpartum depression is a common maternal mental health condition. Existing treatments, such as cognitive behavioral therapy and pharmacological interventions, often face challenges related to cost, accessibility, and acceptability, particularly among breastfeeding mothers.

**Objectives:**

This study aimed to assess the effectiveness of a multidisciplinary health education (iHealth) in reducing postpartum depression and anxiety while enhancing the quality of life among primiparous women.

**Methods:**

This retrospective study, conducted on 192 puerperal women who delivered between January 2020 and December 2021, including 95 in the control group and 97 in the iHealth group. The inclusion criteria were primiparas aged 18-35, with delivery type (vaginal/instrumental/cesarean), 38–42 weeks’ gestation, and infant APGAR ≥8, while those with a history of mental illness were excluded. The sample size (≥64/group) was estimated via independent samples t-test (Cohen’s d=0.5, 80% power, α=0.05). The control group received regular standard health guidance, while the iHealth group additionally received a multidisciplinary health education program via the “iHealth” app (3 times/week, 1h/session, ≥2 months, with reminders), led by a team of doctors, nurses, nutritionists, and psychologists. Blinded psychologists collected data on maternal psychological status-assessed using the Edinburgh Postnatal Depression Scale (EPDS), State-Trait Anxiety Inventory (STAI), and Short Form-12 (SF-12)-at 3 days (baseline), 6 weeks, and 3 months postpartum, with data analyzed using Mann-Whitney, Student’s t or Chi-square tests for group comparisons.

**Results:**

The iHealth group exhibited significantly lower EPDS and STAI scores compared to the control group at both six weeks and three months postpartum. Additionally, the iHealth group demonstrated significantly higher SF-12 scores at these time points, reflecting improved quality of life.

**Conclusions:**

The multidisciplinary health education significantly improved postpartum mental health and enhanced the quality of life for new mothers.

## Introduction

Postpartum depression is a common psychiatric disorder affecting women during the puerperium, typically developing within six weeks after delivery and often resolving spontaneously within three to six months (1, 2). However, in severe cases, symptoms may persist for one to two years, with a recurrence rate of 20%–30% in subsequent pregnancies (3). Postpartum depression is characterized by depression, anxiety, guilt, insomnia, irritability, sadness, confusion, and, in severe instances, suicidal ideation or self-harm (4). Its etiology is multifactorial, involving hormonal fluctuations, childbirth-related pain, and perinatal psychosocial stressors (1). Postpartum depression has serious implications not only for maternal mental health but also for the physical well-being of mothers, the mother-infant bond, and overall family stability (5–7). Its complex pathogenesis includes psychological vulnerability, hormonal shifts, and strained social relationships. Postpartum women often face significant emotional and physical stress due to shifting roles and inadequate social support (8–10). Those with lower psychological resilience are particularly susceptible to mood disturbances that may evolve into depression (11).

Early detection and intervention are crucial. Pregnancy-related education and depression scale screenings can alleviate maternal anxiety, reduce negative emotions, and improve mental well-being (12). As such, health education plays a vital role in preventing the long-term consequences of Postpartum depression for mothers, infants, and families.

Currently, cognitive behavioral therapy (CBT) is the preferred treatment for mild to moderate Postpartum depression, while pharmacological interventions are typically used for severe cases (3). However, CBT is often expensive, and breastfeeding women may be hesitant to use medication (13, 14). These limitations have led to growing interest in alternative interventions, including health education programs (15, 16).

Lack of knowledge is a recognized risk factor for maternal depression (17). Grounding such interventions within a theoretical framework enhances both interpretability and validity of results. The Cox Interaction Model of Client Health Behavior (IMCHB) provides a structured approach for understanding how individual characteristics (e.g., socio-demographics, psychological resilience), emotional and informational support, and healthcare interactions influence postpartum outcomes. This model emphasizes the dynamic interplay between client singularity, client–professional interaction, and health outcomes, thereby offering a socio-psychological lens through which postpartum depression can be analyzed. Guided by the IMCHB, maternal health education provides pregnant women and their families with essential information about pregnancy, childbirth, and postpartum care, while addressing their concerns. This approach promotes healthy behaviors and reduces perinatal complications (18, 19).

To meet the diverse needs of primiparous women—including self-actualization, emotional support, respect, safety, and physical and psychological well-being—we retrospectively evaluated a multidisciplinary health education (iHealth) program. This study aimed to assess its effectiveness in reducing postpartum depression and anxiety, and in improving the quality of life among first-time mothers. By explicitly mapping program components to the IMCHB constructs, the present study ensures that outcome evaluation is theoretically grounded, facilitating assessment of both direct and mediated intervention effects.

Despite increasing awareness and advances in mental health care, no universally effective treatment for Postpartum depression currently exists, and some women continue to experience symptoms even after receiving therapy (20, 21). This underscores the need for comprehensive, preventive, and accessible approaches—such as structured health education—that are theoretically informed and empirically validated to address this widespread condition.

## Methods

### Participants

In this study, a target population of primiparous women delivering at Shijiazhuang Obstetrics and Gynecology Hospital between January 1, 2020, and December 31, 2021, was defined. Eligible women were identified through the hospital’s delivery registry on a rolling basis and screened by trained research nurses during their postpartum hospitalization. All women meeting the inclusion criteria were invited to participate, and those who expressed interest were provided with detailed study information before obtaining written informed consent. A consecutive sampling approach was used to minimize selection bias, enrolling participants in the order they met the criteria until the required sample size was reached.

The study received approval from the Ethics Committee of Shijiazhuang Obstetrics and Gynecology Hospital, and included patients who provided written informed consent. This study was performed in strict accordance with the Declaration of Helsinki, Ethical Principles for Medical Research Involving Human Subjects.

Inclusion criteria:

- Primipara (aged 18–35 years).
- Normal vaginal delivery, instrumental delivery, or caesarean delivery.
- Gestational age of 38–42 weeks.
- Infant APGAR score of eight or higher.

Exclusion criteria:

- Women who were already postpartum.
- Women with a history of mental illness.

**Figure 1:**
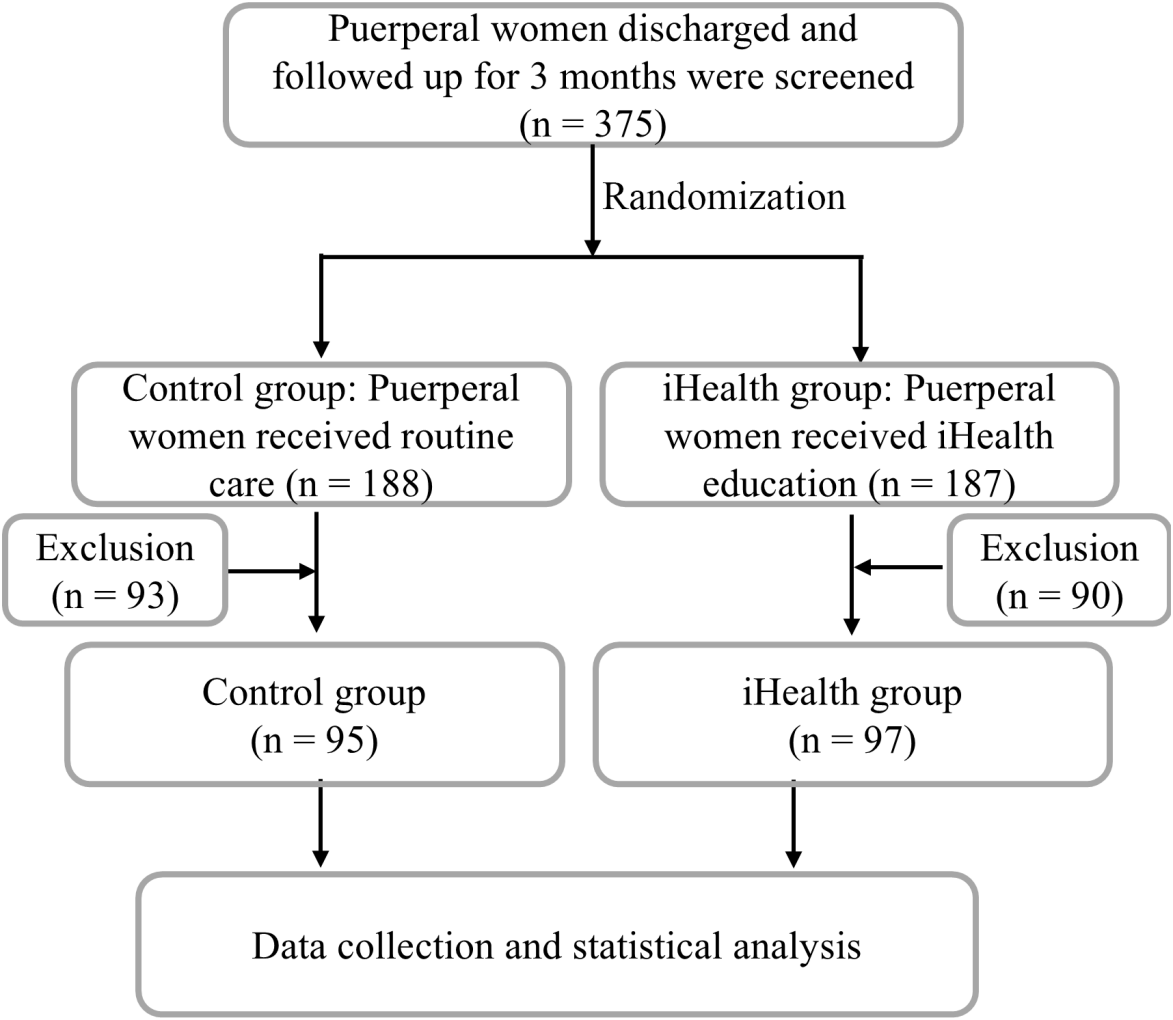
The study flowchart.

A total of 375 puerperal women who were discharged and followed up for three months were initially screened. Among them, 188 received routine care (Control group), and 187 received routine care and a multidisciplinary health (iHealth) education (iHealth group). After applying exclusion criteria, a total of 192 puerperal women were included in the final analysis—95 in the Control group and 97 in the iHealth group (**Figure 1**).

Using an independent samples t-test to examine differences between the control and iHealth groups, we assumed a medium Cohen’s effect size of 0.5 with 80% power and a 0.05 significance level (2-sided). Based on these parameters, a minimum of 64 participants were required for each group.

### Procedures for iHealth group

The mothers in the control group received basic health guidance on a regular basis. In contrast, the mothers in the iHealth group received health education based on the proof-of-concept model in addition to the same routine treatment as the control group. The main components of the procedure included disease education provided by doctors, lifestyle and dietary guidance from nutritionists, and psychological support from counselors.

These education measures primarily relied on the smartphone application iHealth to deliver postpartum-related disease education, lifestyle and dietary guidance, and psychological counseling services to the mothers in the iHealth group. For example, the app offers articles and videos on topics such as stress reduction, nutrition, infant care, and infant vaccination. Users can access iHealth anytime and anywhere.

Additionally, mothers can inquire about issues related to pregnancy, childbirth, and infant health and care through iHealth, with all questions answered by professionals via private messages within the app. The app records parameters such as login frequency and app usage duration.

Mothers in the iHealth group were advised to use iHealth three times a week for one hour each session, for a minimum of two months. If any mother in the iHealth group fails to log in during the study period, she will receive reminders via email or phone.

### Data collection

Baseline assessments were performed within three days of delivery. Subsequent data collection was conducted by professional psychologists, who were blinded to the study, at the sixth week and third month postpartum. Maternal depression, anxiety, and quality of life were assessed at these time points.

### Edinburgh post-birth depression scale

The EPDS was used to assess maternal depression in this study. The EPDS consists of 10 items, with a total score ranging from 0 to 30. The scoring system is as follows: 0–9 points: The vast majority of cases are considered normal. 10–13 points: High-risk group for postpartum depression. 14–30 points: Indicating postpartum depression (22).

### State-trait anxiety inventory

The STAI was used to assess patient anxiety. The STAI consists of 40 items, divided into two subscales that assess different types of anxiety (23).

Items 1 to 20 comprise the State Anxiety Subscale (STAI-Form Y-I, S-AI), with half of the items describing negative emotions and the other half describing positive emotions. This subscale is primarily used to assess an individual’s immediate or recent experience of fear, tension, anxiety, and neuroticism in specific situations or at a particular time.

Items 21 to 40 comprise the Trait Anxiety Subscale (STAI-Form Y-I, T-AI), which assesses relatively stable anxiety and tension-related personality traits. This subscale contains 11 items that describe negative emotions and 9 items that describe positive emotions.

### Short Form-12 scale

The SF-12 was used to assess participants’ quality of life. The SF-12 scale contains a total of 12 questions. This scale consists of twelve questions and is designed to evaluate a patient’s daily activities, self-perception, and social participation. The SF-12 is widely regarded as an important measure of a patient’s overall quality of life (24).

### Statistical methods

All analyses were performed using GraphPad. Prior to analysis, the distribution of continuous variables was assessed using the Shapiro–Wilk test, and homogeneity of variances was checked with Levene’s test to guide the choice of statistical tests. The data are presented as mean ± standard deviation (SD) for continuous variables or n (percentage) for categorical variables. For between-group comparisons of continuous variables that were normally distributed and met variance homogeneity assumptions, the independent samples Student’s t-test was applied. For non-normally distributed continuous variables, the Mann–Whitney U test was used as a non-parametric alternative. Categorical variables were compared using the Chi-square test; when expected cell counts were <5, Fisher’s exact test was employed to ensure valid inference. All statistical tests were two-tailed, and a p-value <0.05 was considered statistically significant.

## Results

### Clinical characteristics of the study participants

Baseline data for 192 patients in the control and iHealth groups were collected prior to the start of the study. As shown in **Table 1**, there were no significant differences between the two groups in terms of age, education, occupation, pregnancy expectations, mode of delivery, and breastfeeding status.

**Table 1:** Characteristics of the participants.

Parameters	Control group (n=95)	iHealth group (n=97)	P value
Age (years)	29.2±3.1	28.7±2.8	0.243
Education level			
≤ high school	70 (73.7%)	67 (69.1%)	0.480
> high school	25 (26.3%)	30 (30.9%)	
Employment			
Employed	56 (58.9%)	55 (56.7%)	0.753
Unemployed	39 (41.1%)	42 (43.3%)	
Planned pregnancy			
Yes	65 (68.4%)	60 (61.9%)	0.340
No	30 (31.6%)	37 (38.1%)	
Type of delivery			
Vaginal delivery	51 (53.7%)	54 (55.6%)	0.533
Instrumental	16 (16.8%)	11 (11.3%)	
Caesarean	28 (29.5%)	32 (33.0%)	
Baby gestational age (weeks)	38.5±2.0	38.8±1.8	0.276
Baby birth weight (g)	3054±811	3133±852	0.511
Breastfeeding one week after birth			
Yes	66 (69.5%)	61 (62.9%)	0.335
No	29 (30.5%)	36 (37.1%)	

Data are presented as mean ± SD or n (%). Student's t test and Chi-square test were used for statistical analysis.

### Change in EPDS and STAI scores between the iHealth and control groups

To illustrate the effect of our proof-of-concept health education on maternal psychological status in the short and long term after delivery, we measured changes in EPDS and STAI scores in the two groups of participants at the sixth week and third month postpartum, respectively.

**Figure 2:**
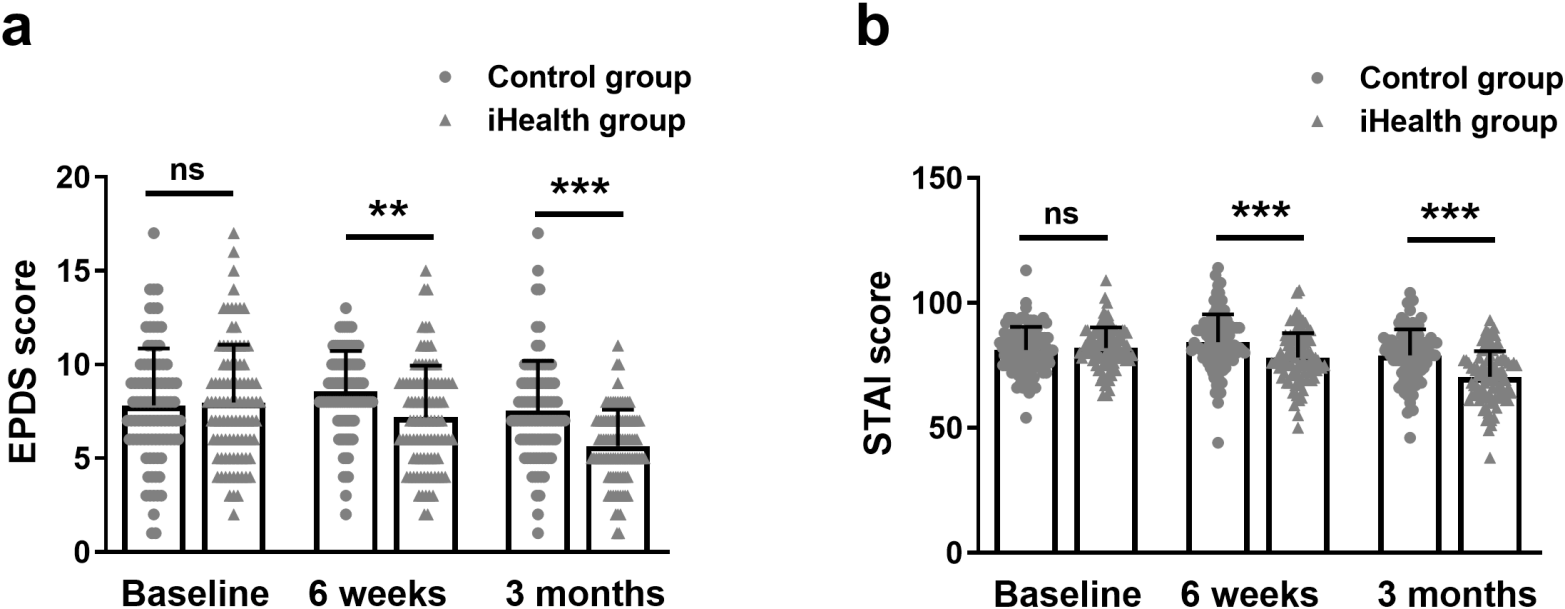
Change in EPDS **(a)** and STAI **(b)** scores between the iHealth and control groups among mothers at baseline, 6 weeks and 3 months postpartum. Data were shown as mean ± SD. **p<0.01, ***p<0.001 from Mann-Whitney test. EPDS, Edinburgh Postnatal Depression Scale; STAI, State-Trait Anxiety Inventory. ns, no significance.

As shown in **Figure 2A**, there was no significant difference in baseline EPDS scores between the control (7.83 ± 3.02) and iHealth groups (7.98 ± 3.08). However, the EPDS scores at the sixth week and third month postpartum were significantly lower in the iHealth group (7.20 ± 2.74, 5.66 ± 1.93) compared to the control group (8.56 ± 2.16, 7.54 ± 2.65). Similarly, STAI scores in the iHealth group (82.00 ± 8.16, 78.13 ± 9.75, 70.41 ± 10.25) were significantly lower than those in the control group (81.18 ± 9.28, 84.29 ± 11.19, 79.08 ± 10.37) at both the sixth week and third month postpartum (**Figure 2B**).

### Change in physical health (SF-12 PCS) and mental health (SF-12 MCS) between the iHealth and control groups

The SF-12 scoring system was used to assess patients’ quality of life. The SF-12 score was divided into two components based on the relevance of the questions: mental health and physical health.

**Figure 3:**
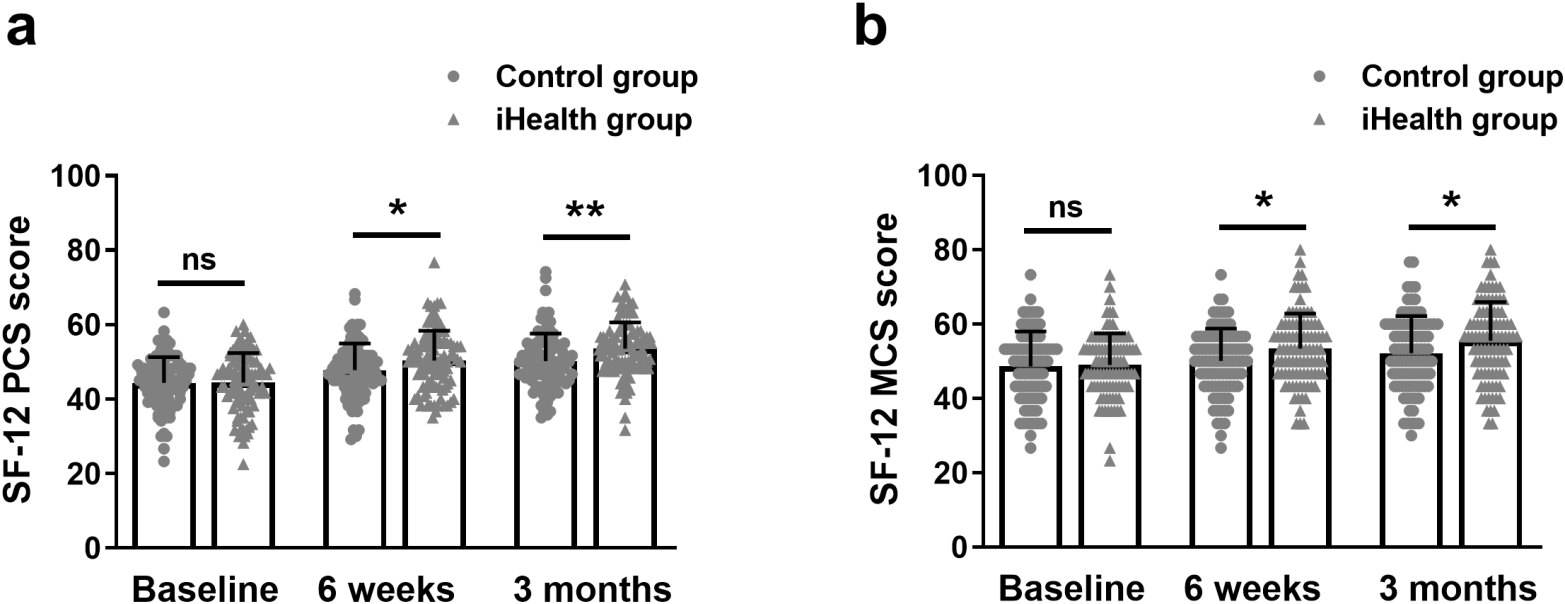
Change in physical health (SF-12 PCS) **(a)** and mental health (SF-12 MCS) **(b)** between the iHealth and control groups among mothers at baseline, 6 weeks and 3 months postpartum. Data were shown as mean ± SD. *p<0.05, **p<0.01 from Mann-Whitney test. SF-12, Rand 12-Item Short Form. ns, no significance.

As shown in **Figure 3A**, the self-assessed physical health scores of the iHealth group (44.48 ± 7.93, 50.37 ± 7.98, 53.54 ± 7.09) were significantly higher than those of the control group (44.39 ± 6.93, 47.76 ± 7.21, 50.18 ± 7.40) at both the sixth week and third month postpartum. Similarly, maternal self-assessed mental health scores in the iHealth group (49.04 ± 8.47, 53.47 ± 9.42, 55.50 ± 10.48) were significantly higher than those in the control group (48.62 ± 9.43, 50.11 ± 8.70, 52.21 ± 9.97) at the same time points (**Figure 3B**).

## Discussion

Postpartum depression can be classified into recovery type, partial improvement type, and aggravation type, depending on symptom severity (25). While most patients experience some relief with treatment, a subset remains in a depressive state that may worsen over time, impairing daily functioning and increasing the risk of adverse outcomes (26). This highlights the critical need for timely and appropriate interventions, such as health education, to support mental health in the postpartum period.

In this study, we evaluated the effectiveness of a structured health education program delivered through a multidisciplinary model involving both doctors and nurses. Unlike routine care, this approach emphasized collaboration and personalized education. Medical staff were trained to consider patients’ psychological perspectives and tailor education to their emotional and cognitive needs. This collaborative model has been shown to enhance patient acceptance of treatment, build trust in medical staff, and improve recovery confidence.

The iHealth intervention integrated practical parenting knowledge with psychological support to help mothers navigate their new roles. It addressed emotional distress and cognitive challenges that often arise during the postpartum period. In developing the program, we also incorporated expertise from nutritionists and psychologists, ensuring a comprehensive and evidence-based approach.

Our findings demonstrated that the iHealth education model significantly reduced maternal anxiety and depression, as reflected in lower EPDS and STAI scores at both six weeks and three months postpartum. Moreover, mothers in the iHealth group showed improved quality of life, with higher SF-12 scores in both physical (PCS) and mental (MCS) domains. These results suggest that our intervention positively influenced both physical and psychological well-being.

These findings are consistent with previous studies showing that structured health education can reduce symptoms of postpartum depression (27–29). For example, digital health education programs have been shown to alleviate postpartum anxiety (30), supporting the utility of our online iHealth platform. Additionally, the incorporation of emotional support aligns with the health behavior interaction model, which emphasizes addressing both cognitive and emotional dimensions of behavior change (31).

Despite promising results, our study has several limitations. First, the sample size was relatively small (fewer than 200 participants), which may limit the generalizability and statistical power of our findings. Second, there remains a lack of standardized scales for assessing postpartum depression, which may influence the consistency of assessments across studies. Third, while we measured anxiety and depression at six weeks and three months postpartum, we did not assess psychological status during pregnancy. Last, protective factors may be confounding elements of the observed results.

To address these gaps, future studies will extend the follow-up period and include prenatal assessments to evaluate changes from pregnancy through the postpartum period. Additionally, we aim to investigate whether implementing prenatal health education can help prevent postpartum depression, offering more comprehensive insights into its long-term benefits.

## Conclusion

In conclusion, the multidisciplinary health education demonstrated significant effectiveness in reducing depression and anxiety scores among primiparas, while also improving their quality of life in this study. These findings suggest that health education is beneficial for mitigating the postpartum depression in pregnant women.

## Data Availability

The original contributions presented in the study are included in the article/Supplementary Material. Further inquiries can be directed to the corresponding author.
